# Idiopathic Spontaneous Intraperitoneal Hemorrhage due to Rupture of Short Gastric Artery Presenting as Massive Gastrointestinal Bleeding: A Rare Case Presentation and Literature Review

**DOI:** 10.7759/cureus.11499

**Published:** 2020-11-16

**Authors:** Yasir Saeed, Zahava Farkas, Sulaiman Azeez

**Affiliations:** 1 Internal Medicine, Lincoln Medical and Mental Health Center, New York, USA; 2 Gastroenterology and Hepatology, Westchester Medical Center, New York, USA; 3 Gastroenterology and Hepatology, Lincoln Medical and Mental Health Center, New York, USA

**Keywords:** idiopathic, intraperitoneal, hemorrhage, abdominal apoplexy, short gastric artery, gastrointestinal bleeding, laparotomy

## Abstract

Idiopathic spontaneous intraperitoneal hemorrhage (ISIH) or abdominal apoplexy is due to the tear of an intra-abdominal visceral vessel spontaneously where no cause can be identified. It is an uncommon but potentially life-threatening condition that generally shows up as a diagnostic dilemma as well as is related to formidable mortality. Among all the reported cases, the extemporaneous tear of short gastric arteries is extremely rare, but it has never been reported to present with massive gastrointestinal bleeding. We report a rare instance of idiopathic spontaneous intraperitoneal hematoma eroding the stomach wall, causing massive gastrointestinal bleeding.

## Introduction

Barber first reported idiopathic spontaneous intraperitoneal hemorrhage (ISIH) in 1909 [1]. It was later on labeled abdominal apoplexy by Green and Powers in 1931 [2], a phenomenon in which there is a tear of an intra-abdominal visceral vessel where we cannot ascertain any cause. It is an uncommon but potentially life-threatening condition that shows up as a diagnostic dilemma associated with formidable mortality [3]. ISIH secondary to the rupture of short gastric arteries is rare, but it has never been reported with massive gastrointestinal bleeding.

## Case presentation

**Figure 1:**
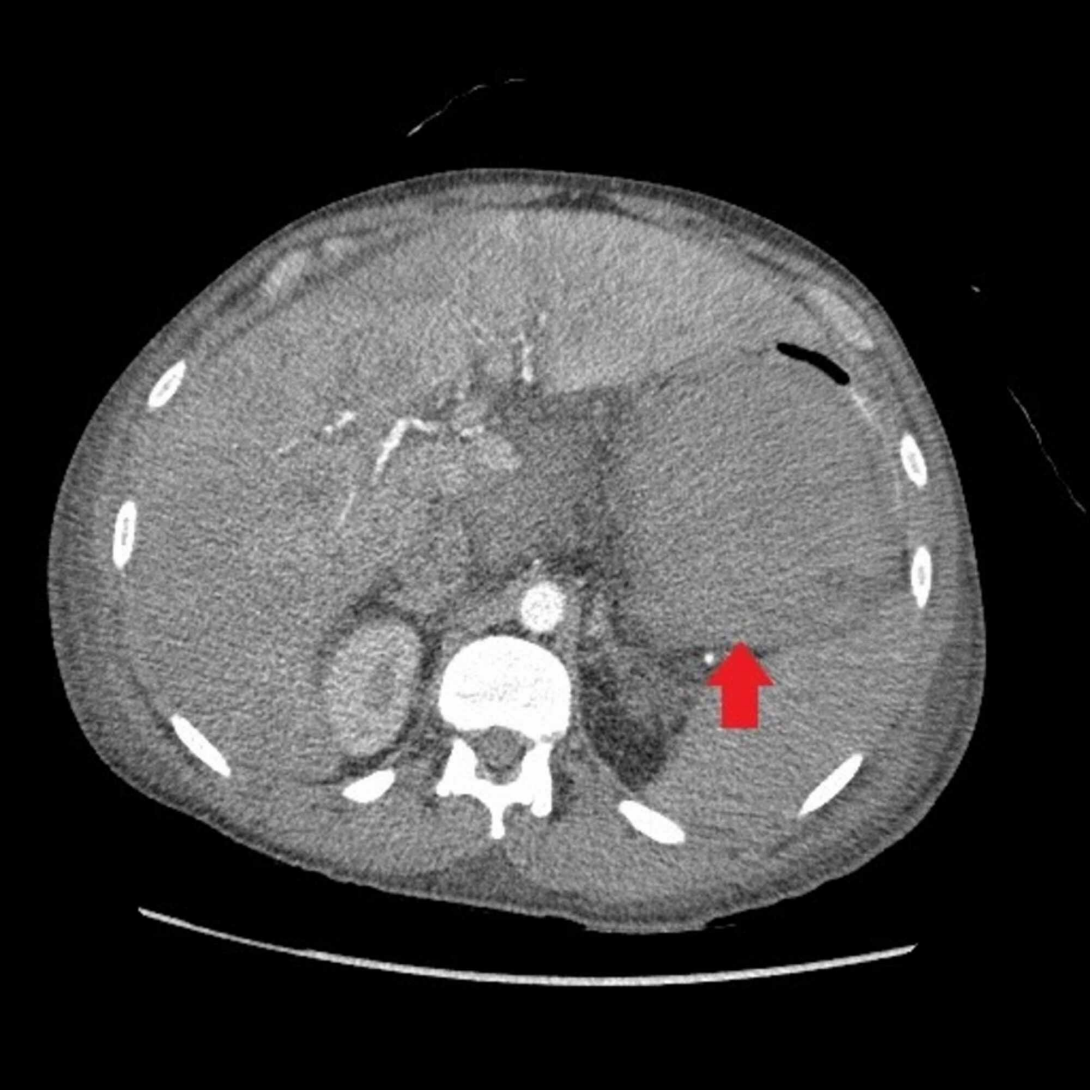
Axial abdominal CTA showing retro gastric intraperitoneal hematoma compressing the stoma CTA: computed tomography angiogram.

**Figure 2:**
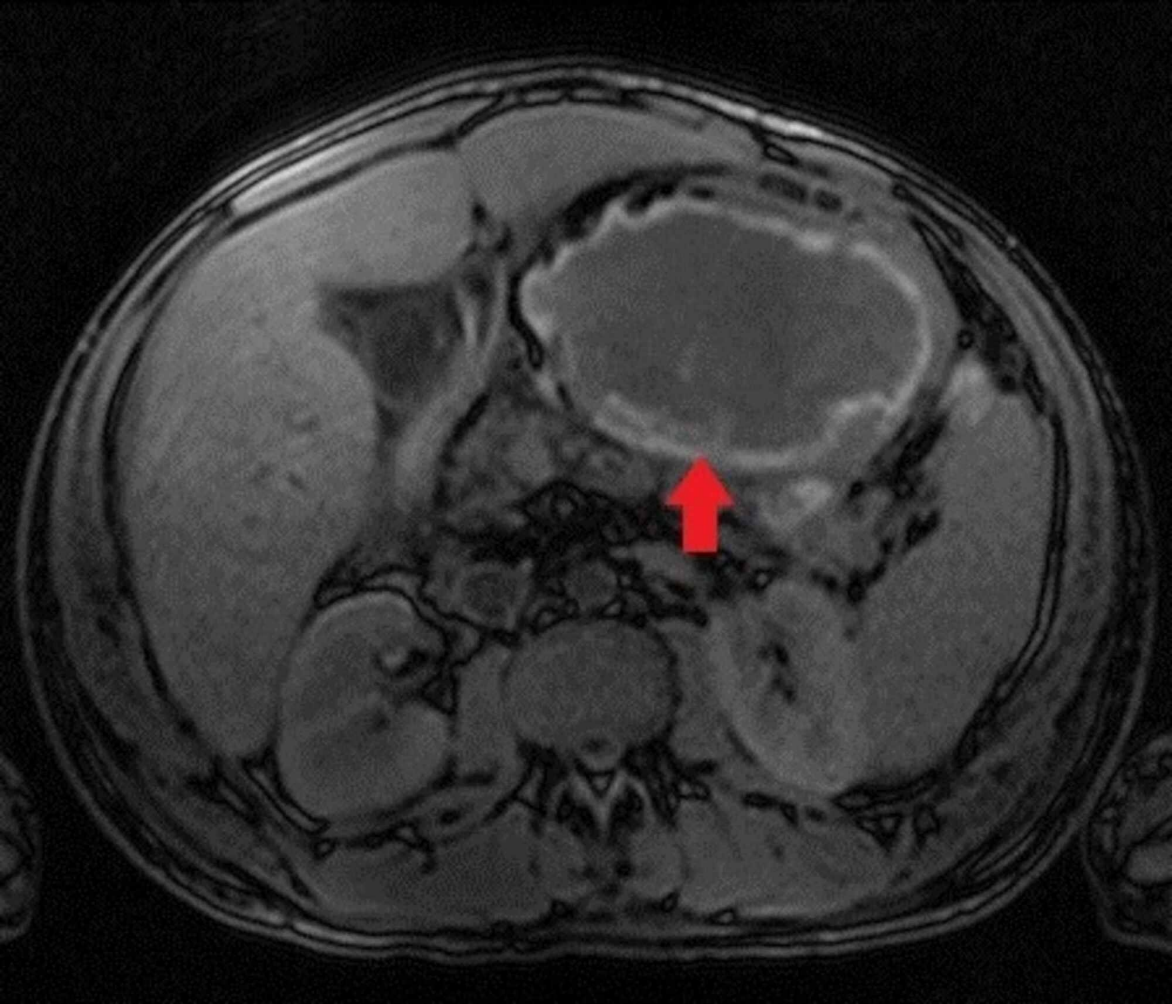
Axial MRI showing advancing encapsulated subacute intraperitoneal hematoma MRI: magnetic resonance imaging.

**Figure 3:**
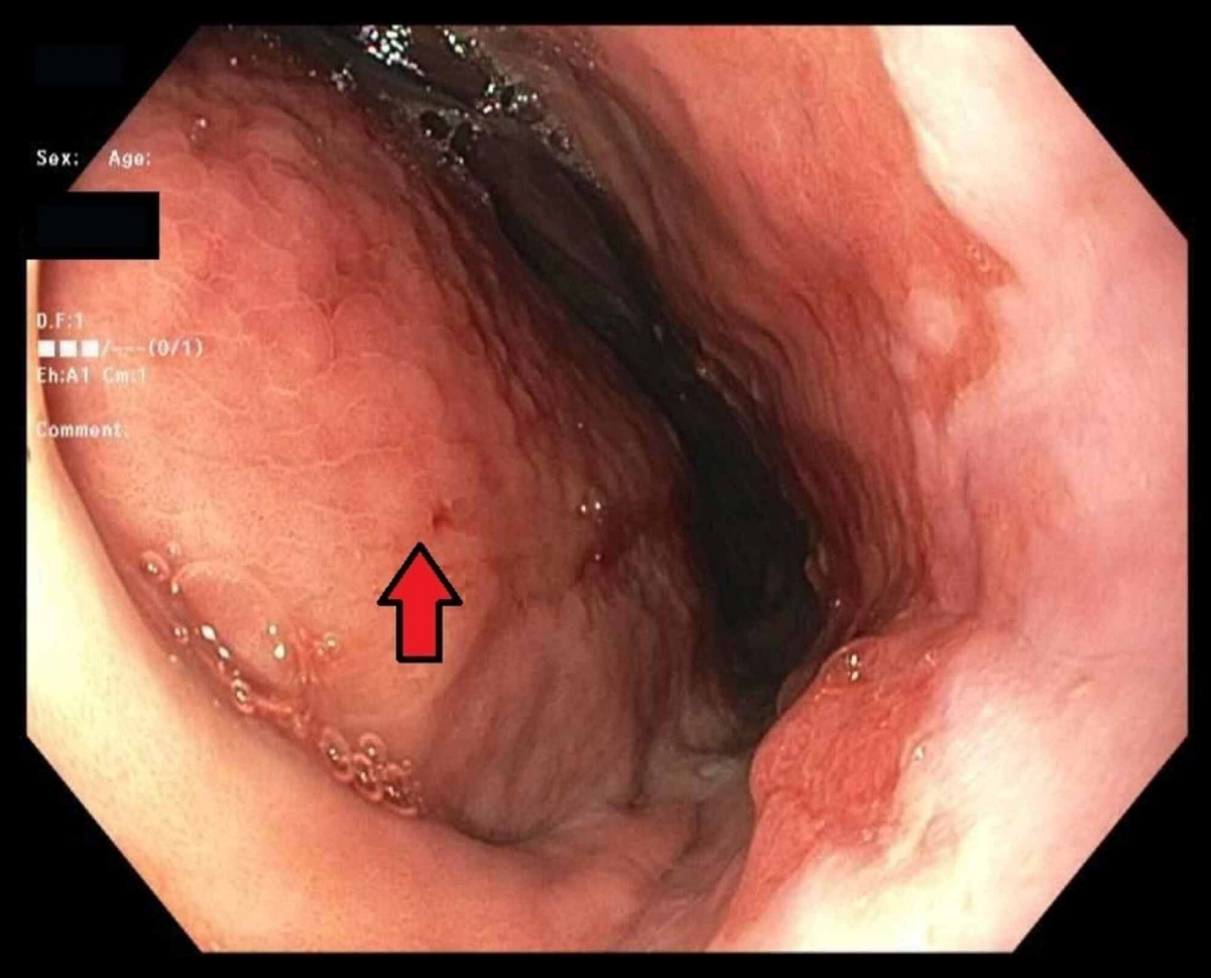
Endoscopic image showing the extrinsic compression of gastric cardia/corpus by hematoma

**Figure 4:**
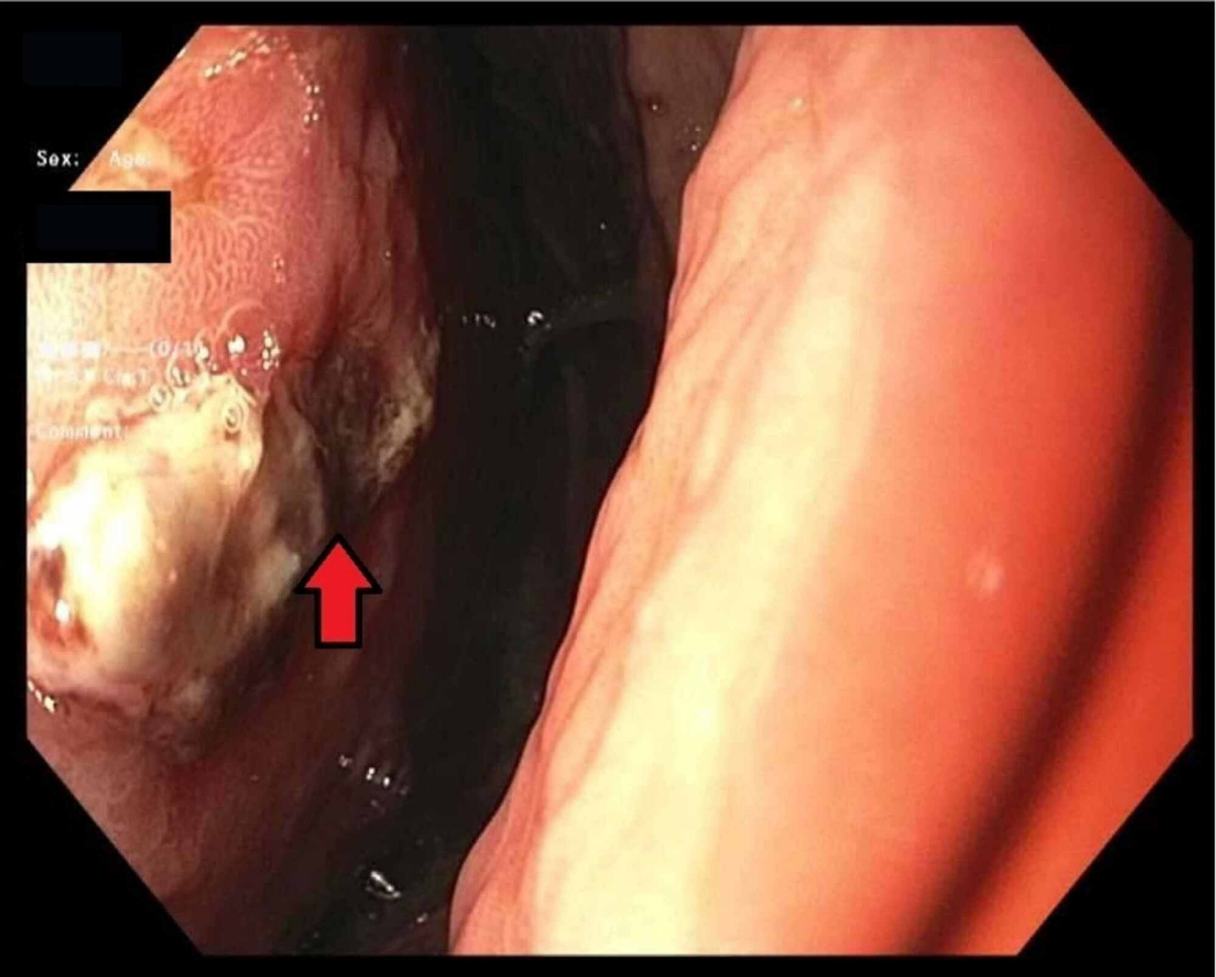
Endoscopic image showing hematoma eroding the gastric cardia on a retroflexed view

A 30-year-old man with a past medical history of Child-Pugh Class B alcoholic cirrhosis, chronic thrombocytopenia, and chronic pancreatitis presenting with constant, sharp, intense, non-radiating diffuse abdominal pain predominantly in the epigastric region for three days associated with nausea and non-biliary, non-bloody vomiting. He denied any trauma, diarrhea, steatorrhea, weight loss, recent NSAID, and alcohol use. Physical examination was worthy of attention for conjunctival pallor, scleral icterus, the abdomen was mildly distended and tender diffusely, worst in the epigastric region with guarding but no rigidity. Initial labs are shown in Table 1. Computed tomography (CT) angiogram of abdomen and pelvis showed hemoperitoneum and retrogastric hematoma in lesser sac, measuring 16.3 × 9 cm^2^ (Figure 1). The surgery team consulted, and a conservative approach was adopted by monitoring patients in an intensive care unit with serial abdominal CT angiogram scans, which showed enlarging hematoma. The hemoglobin/hematocrit and coagulation studies remained stable. Magnetic resonance imaging (MRI), was also done, which showed a large 15 × 10 cm^2^ subacute hematoma in the lesser sac posterior to the stomach but with no source of bleeding identified (Figure 2). Two weeks into his hospital course, the patient exhibited sudden bright red blood per rectum (BRBPR) and hematemesis with drop-in hemoglobin from 9.2 g/dL to 4.1 g/dL. A massive transfusion protocol (MTP) was initiated; the patient received a total of 15 U of packed red blood cells. Emergent esophagogastrodueodoscopy (EGD) was performed and showed extrinsic compression of the intraperitoneal hematoma eroding the gastric cardia/corpus with oozing of blood into the gastric lumen (Figures 3 and 4). There was no spurting, adherent clot, visible vessel, clear edges with a clean base to indicate a gastric mucosal bleeding lesion such as an ulcer or dieulafoy lesion. The patient was taken immediately for exploratory laparotomy. He was found to have an encapsulated hematoma in the lesser sac eroding into the posterior gastric wall; active bleeding from the short gastric arteries was also identified. Evacuation of the hematoma, ligation of the bleeding short gastric arteries, and repair of the gastric wall defect were performed. Successful hemostasis was achieved, and his postoperative course was uneventful. Pathology of encapsulated hematoma showed fibrosis with granulation tissue and fibrin, and non-organized hematoma.

**Table 1:** Initial labs on admission WBC: white blood cells, HGB: hemoglobin, PLT: platelet, ALK PHOS: alkaline phosphatase, ALT: alanine aminotransferase, SGPT: serum glutamic pyruvic transaminase, AST: aspartate aminotransferase, SGOT: serum glutamic oxaloacetic transaminase, BUN: blood urea nitrogen, INR: international normalized ratio, PT: prothrombin time, aPTT: activated partial thromboplastin time.

Laboratory test	Value	Normal range and units
WBC	9.33	4.80 - 10.80 × 10(3)/mcL
HGB	8.4	14.0 - 18.0 g/dL
PLT	55	150 - 450 × 10(3)/mcL
Albumin	3.1	3.5 - 5.2 g/dL
Total bilirubin	5.16	0.20 - 1.20 mg/dL
Direct bilirubin	3.50	0.00 - 0.30 mg/dL
ALK PHOS	169	40 - 130 U/L
ALT (SGPT)	29	£41 U/L
AST (SGOT)	146	£40 U/L
Lipase	35	13 - 60 U/L
BUN	12	6.0 - 23.0 mg/dL
Creatinine	1.57	0.70 - 1.20 mg/dL
INR	1.63	mg/dL
PT	16.8	10.0 - 13.0 seconds
aPTT	35.9	25.0 - 35.3 seconds​

## Discussion

Intra-abdominal hematoma is usually due to ruptured aortic or splanchnic vessels aneurysm, traumatic or iatrogenic vascular injury, neoplasms, autoimmune conditions, and coagulopathy. However, there are rare cases in which no cause is identified and termed ISIH or abdominal apoplexy. Most cases are present in the fifth to seventh decade of life with a 3:1 male to female ratio. In our case, the patient had mild coagulopathy; however, not severe enough to cause spontaneous hemorrhage [4]. ISIH presents itself with a wide variety of signs and symptoms depending on the containment of hemorrhage and the development of a hematoma. Historically, the clinical presentation of ISIH is divided into three phases, an initial phase of mild to moderate acute abdominal pain associated with nausea, vomiting, and anorexia, a latent phase lasting for hours to days without any manifestation, and a terminal phase of rapid progression of symptoms with hemodynamic instability [5-7].

Since initially reported, less than 200 cases of ISIH have been described in the literature [4]. Major risk factors believed to be associated in most cases (80-87%) were the usual causes of atherosclerosis, such as high blood pressure, hyperlipidemia, diabetes, and obesity. The precise mechanism is unknown; however, it is hypothesized to be related to a weak point in the tunica media, leading to a tear with a sudden rise in pressure. Pathology sampling frequently showed disruption of elastic lamellae. The most prevalent bleeding places are the middle colic, left gastric, and hematoma of the mesentery, even though the bleeding vessel is identified in only 62% of laparoscopic inspections [3]. Among all the reported cases of ISIH, the extemporaneous tear of short gastric arteries is very rare. In literature, to the best of our knowledge, we found only 15 cases. Vomiting for any reason is suggested as a common precursor toward short gastric artery rupture. It has been conceptualized that vomiting may cause gastric twisting, which pulls on the gastrosplenic ligament resulting in a short gastric artery rupture [8-10]. We could not find any case reported in the literature in which intraperitoneal hematoma has presented with massive gastrointestinal bleeding. We found rare conditions like aortoenteric fistulae in which there is an abnormal connection between the aorta and the gastrointestinal tract and presents with GI bleeding, but that is usually in the setting of aortic and GI pathology, but in our case, no such kind of pathology identified [11].

Diagnosis is usually made during exploratory laparotomy in the hemodynamically unstable patient, during which detection and ligation of the hemorrhagic vessels are performed with the evacuation of the hematoma. CT scanning is an important diagnostic technique in establishing the site, size of hematoma in hemodynamically stable patients. MRI also plays a useful role in distinguishing between the fluid collection, such as blood and a neoplasm. If no underlying lesion is viewed on CT scanning, then angiography should be performed to detect visceral artery aneurysms; it can also elucidate active bleeding sites. Ultrasound (US) is also valuable to look for an aortic aneurysm, particularly in the hemodynamically unstable patient [6,7,12-14]. Surgical intervention in the form of exploratory laparotomy with a thorough examination of the abdomen to find other causes, evacuation of the hematoma, and ligation of the bleeding vessel described as the definitive treatment option. The prognosis hinges on early diagnosis and intervention. In patients who do not undertake surgery, mortality has historically come closer to 100%. Nevertheless, prompt surgical intervention decreases the mortality to 8.6%, primarily where we can locate and ligate a definite bleeding point [4].

## Conclusions

This case demonstrates the therapeutic and diagnostic challenges associated with ISIH and our currently imperfect understanding of this condition, which requires further research. It is a fatal condition and can present as massive gastrointestinal hemorrhage. Better cognizance of the condition might help improve the prognostication for the patient by promoting early diagnosis and treatment.
